# Raising Awareness of Intraoperative Diagnostic Challenges to Prevent Misdiagnosis and Overtreatment: Laparoscopic Management of Rare Cotyledonoid Dissecting Leiomyoma Mimicking Ovarian Tumour

**DOI:** 10.3390/healthcare13121367

**Published:** 2025-06-06

**Authors:** Kathy Nguyen, Tanushree Rao

**Affiliations:** 1 School of Clinical Medicine, Faculty of Medicine and Health, University of New South Wales, Sydney, NSW 2033, Australia; 2 Minimally Invasive Gynecologic Surgery Unit, Liverpool Hospital, Liverpool, NSW 2170, Australia; dr.tanushreerao@gmail.com

**Keywords:** cotyledonoid dissecting leiomyoma, malignancy, diagnostic challenges, laparoscopy

## Abstract

Cotyledonoid dissecting leiomyoma (CDL), also known as Sternberg tumour, is a rare variant of leiomyoma that can be easily mistaken for a malignant neoplasm on clinical and radiological examination, posing a diagnostic challenge for clinicians. **Background:** Although the tumour can extend to neighbouring organs, it typically does not invade them and is considered benign. Therefore, it is essential to recognise and differentiate this leiomyoma variant from other malignancies to avoid misdiagnosis and overtreatment. **Methods:** This report depicts a unique case of CDL misdiagnosed as an ovarian tumour in a woman in her late 50s with post-menopausal bleeding and pelvic pressure. We initially planned and proceeded with a diagnostic laparoscopy and laparoscopic oophorectomy of the right ovarian mass, during which an intraoperative surprise of a retroperitoneal mass was explored and subsequently biopsied. **Results:** The final histopathology confirmed the presence of the rare fibroid variant CDL. The accompanying surgical video is among the first to feature a laparoscopic surgery of CDL and details the intraoperative findings and laparoscopic resection techniques utilised in this case. **Conclusions:** Given its rarity and non-specific clinical and radiological findings, diagnosing CDL pre-operatively can be challenging. This case prompts recognition and awareness of CDL and highlights the importance of careful consideration of uncommon differential diagnoses and thorough intraoperative exploration, with the goal of preventing the misdiagnosis and, consequently, overtreatment of unknown masses.

## 1. Introduction

Cotyledonoid dissecting leiomyoma (CDL) is an extremely rare variant of leiomyoma which can be locally invasive and extend to neighbouring organs such as the bladder, rectum, and fallopian tubes. It typically does not invade them and is considered a benign tumour with good prognosis [1,2,3]. However, CDL can easily be mistaken for a malignant neoplasm on clinical and radiological examination, posing a diagnostic challenge for clinicians [4]. Therefore, it is essential to recognise and differentiate this variant from other malignancies to avoid misdiagnosis and overtreatment, especially in women who wish to preserve their fertility. Herein, we report a rare case of CDL misdiagnosed pre-operatively as an ovarian tumour and explore the diagnostic challenges associated with CDL as well as the approach for its laparoscopic removal.

## 2. Case Presentation

**Figure 1:**
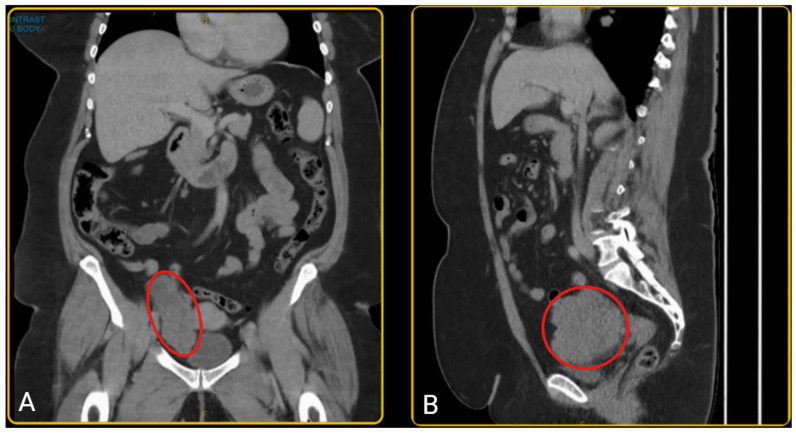
(**A**) The coronal view of the pelvic CT scan; (**B**) the sagittal view of the pelvic CT scan showing a mass in the right lower quadrant of the abdomen, anteromedial to the iliac vessels and posterior to the bladder, consistent with a right adnexal mass (outlined in red). The mass measures 103 × 98 × 50 mm, with a well-demarcated, irregular border and has a heterogenous composition with varying internal density.

A woman in her late 50s presented with a history of post-menopausal bleeding and pelvic pressure. A pelvic ultrasound and CT scan showed a right adnexal mass measuring 103 × 98 × 50 mm (Figure 1). The mass had an irregular wall and a solid component. However, her tumour markers were normal (CA 125 = 13; Ca 19-9 = 22; CEA = 2.5; AFP = 7; RMI = 111). She was pre-emptively diagnosed with an ovarian tumour; however, since the diagnosis was not established, pre-operative biopsy was not performed due to the risk of tumour seeding and technical challenges. The patient was planned for hysteroscopy, endometrial sampling, laparoscopy +/− bilateral salpingo-oophorectomy after a detailed discussion of the diagnostic uncertainty and potential histopathological findings. Given her age, post-menopausal status, and clinical presentation, she understood the rationale for surgical intervention and provided informed consent for the proposed plan.

Endometrial cancer was ruled out with a hysteroscopy dilation and curettage and polypectomy immediately prior to the laparoscopy and oophorectomy, revealing an atrophic endometrium and a benign endometrial polyp.

**Figure 2:**
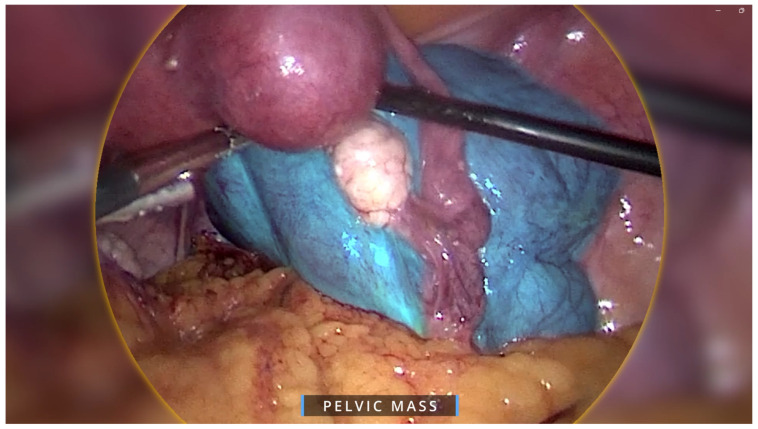
Intraoperative image showing overall view of pelvic mass in blue overlay. The mass was attached to the uterus laterally, anteriorly extending up to the paravesical space, laterally to the external iliac vessels, and posteriorly in the pararectal space.

**Figure 3:**
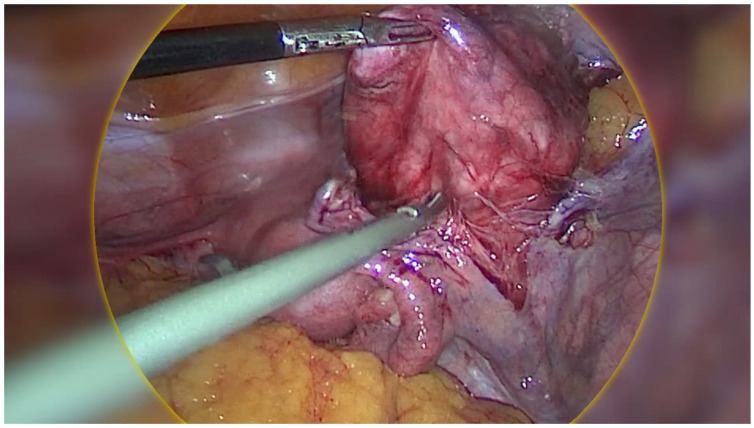
Intraoperative image of cotyledonoid dissecting leiomyoma (CDL) which grossly resembles cotyledons of the placenta.

Regarding the ovarian tumour, the patient underwent a diagnostic laparoscopy using a 4-port laparoscopic technique with an ipsilateral configuration, during which we were met with an intraoperative surprise. A retroperitoneal pelvic mass of unknown origin was found and further explored. The mass measured around 8 × 8 cm and was found to be attached to the uterus laterally, anteriorly extending up to the paravesical space, laterally to the external iliac vessels, and posteriorly in the pararectal space (Figure 2). Initially, the mass was thought to be a fibroid; however, on palpation the consistency of the mass was different to that of a usual fibroid and was soft, almost jelly-like. The gross features also resembled cotyledons of the placenta (Figure 3). Due to the unique pattern of growth and inconclusive appearance of the retroperitoneal mass, we consulted with a gynaecology oncologist intraoperatively via telehealth and proceeded with their recommendation to perform an excisional biopsy.

The mass’s jelly-like consistency and attachment to the iliac vessels laterally required us to develop a plane between the lymph nodes of the pelvic side wall and the tumour in order to prevent injury to the iliac vessels. Once the mass was carefully dissected and fully mobilised, it was placed in a laparoscopic bag and removed from the abdominal cavity. Surprisingly, the bag was able to be extracted through the 15 mm port site via the umbilicus without any extension due to the jelly-like nature of the mass.

The excised mass was sent for testing, which revealed a CDL, a rare variant of leiomyoma. Macroscopically, it appeared to have a smooth peritonealised surface with a solid, lobulated, white, fibrotic cut surface, with the lobules ranging from 2 to 22 mm in diameter. Areas of haemorrhage and increased vascularity were also noted. Histopathological examination of the mass demonstrated moderate vascularity and prominent oedematous areas separating the lobules of smooth muscle. Immunohistochemical analysis showed that the smooth muscle was desmin positive, S100 negative, ruling out endometrial stromal tumours as well as melanocytic and neural tissue lesions, respectively. There was no atypia of the smooth muscle cells, mitotic activity, or evidence of necrosis. No malignant cells were seen in the peritoneal washings sent for cytology.

The patient recovered well and was discharged the following day without complications. She was informed of the benign histopathology findings at her 6-week follow-up appointment and expressed relief and satisfaction with the outcome, as well as appreciation for the surgical restraint shown and preservation of her ovaries. During her follow-up in a year the patient was well with no evidence of clinical symptoms or disease progression.

## 3. Discussion

CDL, also known as Sternberg tumour, was first reported by Roth et al. It has been reported in women aged 23–73 years old, with most cases occurring in women aged 30–50, where common symptoms include lower abdominal pain and abnormal uterine bleeding [1,2]. The pathogenesis of CDL is not well understood, but it is believed to arise from a stem cell population in the myometrium [1]. Table 1 summarises CDL cases reported in the literature, including patient age, clinical presentation, tumour size, and management received.

CDL is a rare histological variant of leiomyoma (benign uterine smooth muscle tumour) with less than 70 cases reported in the English literature [2]. Other well-recognised variants within the leiomyoma spectrum are summarised in Table 2 [35,36]. Importantly, CDL should not be confused with leiomyomas exhibiting disseminated, intravascular, or metastasizing growth, such as disseminated peritoneal leiomyomatosis (DPL), intravenous leiomyomas, or benign metastasizing leiomyomas, as it is a distinct entity which does not invade surrounding structures and lacks vascular and extrauterine invasion [35,36]. Another important differential to consider is smooth muscle tumours of uncertain malignant potential (STUMP) which are a clinically and pathologically heterogenous group of uterine smooth muscle tumours [37]. These tumours exceed the criteria for a leiomyoma and demonstrate malignant potential to develop into a low-grade leiomyosarcoma in a minority of cases yet satisfy insufficient criteria for leiomyosarcoma (marked nuclear atypia, high mitotic rate of >10 mitoses per 10 high-power fields, and tumour cell necrosis) [38]. More often, the rarity and non-specific, alarming features of CDL lead to its misdiagnosis for more sinister differentials including ovarian tumours, leiomyosarcomas, and endometrial stromal sarcoma, even though CDL is benign and carries a good prognosis [34]. This poses a diagnostic challenge for clinicians, especially in the pre-operative setting, and can lead to overtreatment. Therefore, recognition and awareness of CDL is essential to prevent such misdiagnosis and overtreatment.

**Figure 4:**
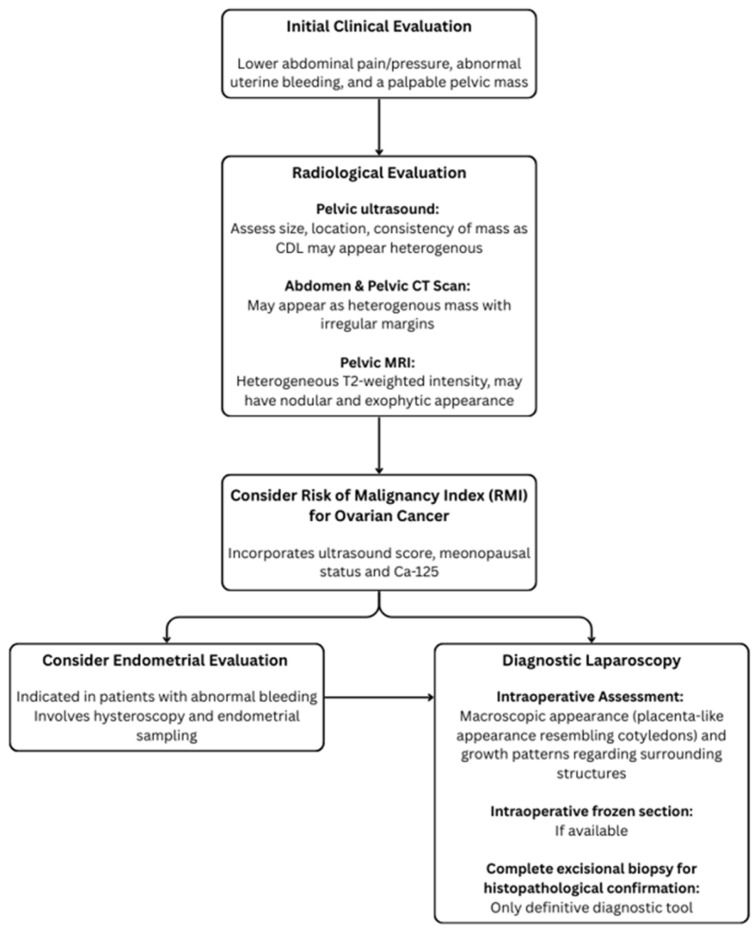
Structured diagnostic pathway for suspected cotyledonoid dissecting leiomyoma (CDL).

Pre-operative diagnostic techniques for CDL often yield an inconclusive or incorrect diagnosis. The diagnostic work-up for CDL is summarised in Figure 4 and begins with clinical evaluation and screening for signs and symptoms similar to those of typical leiomyomas, including lower abdominal pain/pressure, abnormal uterine bleeding, and a palpable pelvic mass. Radiological evaluation can include pelvic ultrasound and CT as well as MRI, where CDL often presents as heterogenous mass, raising concerns of malignancies such as leiomyosarcomas. However, to date, these imaging modalities cannot clearly differentiate malignant differentials from CDL [39]. An incisional core biopsy can be valuable for pre-operative diagnosis but is limited by sampling errors and insufficient information, where the biopsy may not capture the representative tissue, especially in heterogeneous tumours. Additionally, the risk of tumour seeding discourages the use of incisional core biopsies in situations where the diagnosis has not been confirmed [40]. In our case, since the pre-operative diagnosis was not established, an incisional core biopsy was not applicable.

CDL is often found incidentally in the intraoperative setting based on its unusual macroscopic appearance and pattern of growth. The size range has been reported from 25 mm to 410 mm in diameter, although most fall between 100 and 200 mm (average = 148.2 mm), with our case representing a smaller CDL mass. This could be attributed to the effect of hormone depletion in menopause, leading to smaller-sized lesions with advancing age, as described by Buonomo et al. [41]. Macroscopically, CDL typically appears as a soft, jelly-like, multinodular, grape-like mass that is red-brown in colour, and has a placenta-like appearance resembling cotyledons. Despite being able to dissect its way through adjacent organs such as the bladder, rectum, and fallopian tubes, CDL does not invade these surroundings structures [4,13,42]. Thus, despite its atypical gross appearance and pattern of growth which can raise suspicions of sarcomas, CDL does not metastasise and carries a good prognosis. This demonstrates how careful intraoperative exploration and recognition of CDL based on its unique features can prompt consideration of alternative diagnoses rather than malignancies, hence guiding intraoperative decision-making to avoid overtreatment.

When in doubt, intraoperative frozen sections may be considered as they can provide immediate information to guide surgical decisions on whether a more conservative approach is sufficient or if more extensive surgery is required but may have limitations in accuracy due to the presence of artefacts and can prolong surgery [43]. In this case, the facility where the surgery was performed did not have intraoperative frozen section capabilities. Instead, we consulted with a gynaecologic oncologist, and decided to proceed with an excisional biopsy instead of the initial intended oophorectomy, thus sparing the patient from unnecessary overtreatment.

Ultimately, histopathological examination remains the only diagnostic tool for a definitive diagnosis of CDL, whereby it is uniquely characterised by the presence of irregular nodular dissections of smooth muscle cells within the myometrium [13].

The treatment options for CDL depend on several factors, including the patient’s age, symptoms, and desire for future fertility. In many cases, observation may be appropriate, especially in post-menopausal women who are asymptomatic. For symptomatic women, surgical resection may be recommended [34]. Although surgical management of CDL in the literature has mostly involved total abdominal hysterectomy +/− bilateral salpingo-oophorectomy, good outcomes have been reported for more conservative approaches including total tumour excisional biopsy/resection and myomectomy, thus they may be preferred, especially in patients desiring fertility-sparing techniques [41].

Overall, CDL carries a good prognosis with most patients remaining disease-free on long-term follow up, and no confirmed cases of recurrence, malignant transformation, or metastasis have been reported [41].

## 4. Conclusions

CDL is a rare, benign variant of leiomyoma with a good prognosis. However, it can be easily misdiagnosed as ovarian tumours, posing a diagnostic challenge for clinicians. This case and the accompanying surgical video is among the first to feature a laparoscopic surgery of CDL and highlights the importance of thorough intraoperative exploration and careful consideration of uncommon differential diagnoses for ovarian tumours. Recognition and awareness of CDL by clinicians and pathologists can prevent misdiagnosis for a more sinister malignant condition, and consequently overtreatment, especially in fertility-seeking women. Further research may be required to gain a more comprehensive understanding of this pathological variant and develop more robust pre- and intraoperative diagnostic techniques.

## Figures and Tables

**Table 1:** Reported cases of cotyledonoid dissecting leiomyoma in the literature.

Reference	Age	Clinical Presentation	Tumour Maximum Dimension (mm)	Management
Roth et al. [1]	39	Pelvic mass	103	TH + BSO
	41	Abnormal uterine bleeding	100	
	23	Pelvic mass	250	
David et al. [5]	65	Abnormal uterine bleeding	150	TH
	48	Uterine prolapse	120	
Brand et al. [6]	24	Abdominal mass	NS	M
Roth & Reed [7]	46	Pelvic mass	340	TH + BSO
Kim et al. [8]	26	Incidental	120	M
Cheuk et al. [9]	55	Abnormal uterine bleeding	100	TH + BSO
Stewart et al. [10]	58	Pelvic mass	164	TH + BSO + O
Gurbuz et al. [11]	67	Incidental	100	TH + BSO
Jordan et al. [12]	46	Adnexal mass	220	H +/− BSO
	46	Pelvic mass	200	
	46	Pelvic mass	100	
	46	Pelvic mass	180	
	36	Abnormal uterine bleeding	130	
	34	Uterine mass	180	M
Saeed et al. [13]	27	Pelvic mass	410	TH + BSO
Maimoon et al. [14]	40	Urinary retention	100	TH + SO
Shelekhova et al. [15]	73	Uterine mass	80	TH + BSO
Weissferdt et al. [16]	52	Abnormal uterine bleeding	62	TH + S
Adedipe et al. [17]	52	Menorrhagia	NS	TH + O
Raga et al. [18]	33	Abdominal pain	60	UPA + M
Preda et al. [19]	41	Uterine mass	90	TH + ovariectomy
Fukunaga et al. [20]	56	Constipation	300	TH + BSO
	47	Abdominal pain	260	
	36	Abnormal uterine bleeding	40	
	35	Abdominal pain	180	
Gezginç K et al. [21]	57	Pelvic pain	45	TH + BSO
Agarwal et al. [22]	52	Abnormal uterine bleeding	85	TH
Tanaka et al. [23]	36	Incidental	100	M
Onu et al. [24]	50	Incidental	100	RH + BSO + O + A
Blake et al. [25]	56	Abnormal uterine bleeding	300	RH + BSO + O
Shimizu et al. [26]	40	Abnormal uterine bleeding	100	TH + BS
Xu et al. [2]	55	Pelvic mass	60	TH + BSO
	43	Pelvic mass	30	
	37	Plevic mass	300	
	48	Abdominal pain	67	
Lenz et al. [27]	64	Pelvic pain, loss of R kidney function	NS	TH + R
Rocha et al. [28]	38	Abdominal pain and menorrhagia	250	TH + BSO
Parker et al. [29]	39	Abdominal mass	NS	M
Kawashita et al. [30]	45	Pelvic mass	220	TH + SO
Yadav et al. [31]	65	Pelvic pain, urinary retention, abnormal uterine bleeding	NS	TH
Dong et al. [32]	39	Pelvic mass	73	TH + BSO
Robichaud et al. [33]	29	Abdominal pain and menorrhagia	110	M
Chahkandi et al. [34]	55	Abnormal uterine bleeding	140	TH + BSO
Abreu et al. [4]	44	Abdominal pain	100	TH + SO + S

Abbreviations: NS—not specified; TH—total hysterectomy; M—myomectomy; BSO—bilateral salpingo-oophorectomy; SO—unilateral salpingo-oophorectomy; UPA—ulipristal acetate; BS—bilateral salpingectomy; A—appendicectomy; RH—radical hysterectomy; O—omentectomy; S—unilateral salpingectomy; R—unilateral parametrectomy, partial resection of the bladder wall and resection of the distal part of the ureter.

**Table 2:** Summary of histological leiomyoma variants.

Leiomyoma Variants	Histological Features
Cellular leiomyoma	Increased cellularity compared to surrounding myometrium Lacks nuclear atypia, elevated mitotic activity or necrosis
Mitotically active leiomyoma	6–14 mitoses per 10 high-power fields Lacks nuclear atypia or necrosis
Leiomyoma with bizarre nuclei	Marked nuclear atypia (including multinucleation and hyperchromasia) Lacks elevated mitotic activity or necrosis
Epithelioid leiomyoma	Rounded or polygonal cells with eosinophilic or clear cytoplasm arranged in nests or cords Lacks nuclear atypia, mitosis, or necrosis
Myxoid leiomyoma	Hypocellular tumour with abundant myxoid stroma Lacks nuclear atypia, mitosis, or necrosis

## Data Availability

The original contributions presented in this study are included in the article/Supplementary Materials. Further inquiries can be directed to the corresponding author.

## Supplementary Materials

The following supporting information can be downloaded at: https://www.mdpi.com/article/10.3390/healthcare13121367/s1, Surgical video depicting the case presentation and laparoscopic discovery and excision of the rare cotyledonoid dissecting leiomyoma (CDL), initially misdiagnosed as an ovarian tumour.

## References

[1] Roth L.M., Reed R.J., Sternberg W.H. (1996). Cotyledonoid dissecting leiomyoma of the uterus. The Sternberg tumor. Am. J. Surg. Pathol..

[2] Xu T., Wu S., Yang R., Zhao L., Sui M., Cui M., Chang W. (2016). Cotyledonoid dissecting leiomyoma of the uterus: A report of four cases and a review of the literature. Oncol. Lett..

[3] Jamal I., Gupta R.K., Sinha R.K., Bhadani P.P. (2019). Cotyledonoid dissecting leiomyoma: An uncommon form of a common disease. Obstet. Gynecol. Sci..

[4] Abreu R.F., Bovolim G., Baiocchi G., De Brot L. (2023). Cotyledonoid dissecting leiomyoma of the uterus: A gross and radiologic malignancy mimicker. Int. J. Gynecol. Cancer.

[5] David M.P., Homonnai T.Z., Deligdish L., Loewenthal M. (1975). Grape-like leiomyomas of the uterus. Int. Surg..

[6] Brand A.H., Scurry J.P., Planner R.S., Grant P.T. (1995). Grape-like leiomyoma of the uterus. Am. J. Obstet. Gynecol..

[7] Roth L.M., Reed R.J. (1999). Dissecting leiomyomas of the uterus other than cotyledonoid dissecting leiomyomas: A report of eight cases. Am. J. Surg. Pathol..

[8] Kim M.J., Park Y.K., Cho J.H. (2002). Cotyledonoid dissecting leiomyoma of the uterus: A case report and review of the literature. J. Korean Med. Sci..

[9] Cheuk W., Chan J.K., Liu J.Y. (2002). Cotyledonoid leiomyoma: A benign uterine tumor with alarming gross appearance. Arch. Pathol. Lab. Med..

[10] Stewart K.A., Ireland-Jenkin K., Quinn M., Armes J.E. (2003). Cotyledonoid dissecting leiomyoma. Pathology.

[11] Gurbuz A., Karateke A., Kabaca C., Arik H., Bilgic R. (2005). A case of cotyledonoid leiomyoma and review of the literature. Int. J. Gynecol. Cancer.

[12] Jordan L.B., Al-Nafussi A., Beattie G. (2002). Cotyledonoid hydropic intravenous leiomyomatosis: A new variant leiomyoma. Histopathology.

[13] Saeed A.S., Hanaa B., Faisal A.S., Najla A.M. (2006). Cotyledonoid dissecting leiomyoma of the uterus: A case report of a benign uterine tumor with sarcoma-like gross appearance and review of literature. Int. J. Gynecol. Pathol..

[14] Maimoon S., Wilkinson A., Mahore S., Bothale K., Patrikar A. (2006). Cotyledonoid leiomyoma of the uterus. Indian. J. Pathol. Microbiol..

[15] Shelekhova K.V., Kazakov D.V., Michal M. (2007). Cotyledonoid dissecting leiomyoma of the uterus with intravascular growth: Report of two cases. Virchows Arch..

[16] Weissferdt A., Maheshwari M.B., Downey G.P., Rollason T.P., Ganesan R. (2007). Cotyledonoid dissecting leiomyoma of the uterus: A case report. Diagn. Pathol..

[17] Adedipe T.O., Vine S.J. (2010). Dissecting Cotyledonoid Leiomyoma: A Rare Cause of Chronic Intractable Menorrhagia (Not Amenable to Medical Treatment). Case Report. Eur. J. Gynaecol. Oncol..

[18] Raga F., Sanz-Cortés M., Casañ E.M., Burgues O., Bonilla-Musoles F. (2009). Cotyledonoid dissecting leiomyoma of the uterus. Fertil. Steril..

[19] Preda L., Rizzo S., Gorone M.S., Fasani R., Maggioni A., Bellomi M. (2009). MRI features of cotyledonoid dissecting leiomyoma of the uterus. Tumori.

[20] Fukunaga M., Suzuki K., Hiruta N. (2010). Cotyledonoid dissecting leiomyoma of the uterus: A report of four cases. APMIS.

[21] Gezginç K., Yazici F., Selimoğlu R., Tavli L. (2011). Cotyledonoid dissecting leiomyoma of the uterus with intravascular growth in postmenopausal woman: A case presentation. Int. J. Clin. Oncol..

[22] Agarwal R., Radhika A.G., Malik R., Radhakrishnan G. (2010). Cotyledonoid leiomyoma and non-descent vaginal hysterectomy. Arch. Gynecol. Obstet..

[23] Tanaka H., Toriyabe K., Senda T., Sakakura Y., Yoshida K., Asakura T., Taniguchi H., Nagao K. (2013). Cotyledonoid dissecting leiomyoma treated by laparoscopic surgery: A case report. Asian J. Endosc. Surg..

[24] Onu D.O., Fiorentino L.M., Bunting M.W. (2013). Cotyledonoid Dissecting Leiomyoma as a Possible Cause of Chronic Lower Back Pain. BMJ Case Rep..

[25] Blake E.A., Cheng G., Post M.D., Guntupalli S. (2015). Cotyledonoid dissecting leiomyoma with adipocytic differentiation: A case report. Gynecol. Oncol. Rep..

[26] Shimizu A., Tanaka H., Iwasaki S., Wakui Y., Ikeda H., Suzuki A. (2016). An unusual case of uterine cotyledonoid dissecting leiomyoma with adenomyosis. Diagn. Pathol..

[27] Lenz J., Chvátal R., Konečná P. (2020). Dissecting leiomyoma of the uterus with unusual clinical and pathological features. Ceska Gynekol..

[28] Rocha A.C., Oliveira M., Luís P., Nogueira M. (2018). Cotyledonoid dissecting leiomyoma of the uterus: An unexpected diagnosis after delivery. Acta Med. Port..

[29] Parker W.H., Turner R., Schwimer S., Foshag L. (2020). Massive Cotyledenoid Leiomyoma Treated with Uterine-Conserving Surgery. FS Rep..

[30] Kawashita S., Nonoshita A., Iwasaki K., Nakayama D. (2024). Cotyledonoid dissecting leiomyoma: A rare benign uterine tumor mimicking malignancy. Clin Pathol..

[31] Yadav A., Raychaudhuri S., Kaur L., Bhardwaj M. (2024). Cotyledonoid dissecting leiomyoma: A rare case report. Caspian J. Reprod. Med..

[32] Xue Dong R. (2024). Cotyledonoid dissecting leiomyoma mimicking ovarian malignancy: A case report and literature review. J. Minim Invasive Gynecol..

[33] Robichaud S., Wong J., Ouallouche K., Bleau N., Rahimi K. (2025). Dissecting “Cotyledonoid” Leiomyoma Involved by Adenomyosis. Int. J. Surg. Pathol..

[34] Chahkandi M., Ataei M., Bina A.R., Mozayani F., Fanoodi A. (2023). Cotyledonoid dissecting leiomyoma of the uterus: A case report and review of the literature. J. Med. Case Rep..

[35] WHO (2020). Classification of Female Genital Tumours.

[36] Arleo E.K., Schwartz P.E., Hui P., McCarthy S. (2015). Review of leiomyoma variants. Am. J. Roentgenol..

[37] Tinelli A., D’Oria O., Civino E., Morciano A., Hashmi A.A., Baldini G.M., Stefanovic R., Malvasi A., Pecorella G. (2023). Smooth muscle tumor of uncertain malignant potential (STUMP): A comprehensive multidisciplinary update. Medicina.

[38] National Organization for Rare Disorders (NORD) (2023). Leiomyosarcoma—Symptoms, Causes, Treatment. RareDiseases.org. https://rarediseases.org/rare-diseases/leiomyosarcoma/.

[39] Asghari K.M., Tabrizi A.D., Madani P.S. (2024). Unraveling the mystery of uterine cotyledonoid dissecting leiomyoma: A case report. Eur. J. Gynaecol. Oncol..

[40] Yarram S.G., Nghiem H.V., Higgins E., Fox G., Nan B., Francis I.R. (2007). Evaluation of imaging-guided core biopsy of pelvic masses. Am. J. Roentgenol..

[41] Buonomo F., Bussolaro S., Fiorillo C.d.A., Giorda G., Romano F., Biffi S., Ricci G. (2021). The management of the cotyledonoid leiomyoma of the uterus: A narrative review of the literature. Int. J. Environ. Res. Public Health.

[42] Fernandez K., Cheung L., Taddesse-Heath L. (2022). Cotyledonoid dissecting leiomyoma: A rare variant of leiomyoma of the uterus. Cureus.

[43] Karki D., Shrestha G., Joshi S.L. (2024). A case of cotyledonoid dissecting leiomyoma with associated disseminated peritoneal leiomyomatosis: The significance of frozen section in identification of this unusual entity. Cureus.

